# The effectiveness of antibacterial therapeutic clothing based on silver or chitosan as compared with non-antibacterial therapeutic clothing in patients with moderate to severe atopic dermatitis (ABC trial): study protocol for a pragmatic randomized controlled trial

**DOI:** 10.1186/s13063-021-05836-y

**Published:** 2021-12-11

**Authors:** Aviël Ragamin, Karin B. Fieten, Ron A. Tupker, Jill de Wit, Minke M. F. van Mierlo, Marieke S. Jansen, Madelon B. Bronner, Renske Schappin, Frank H. J. Schuren, Margreet L. E. Romeijn, Bernd W. M. Arents, Suzanne Polinder, Marlies de Graaf, Thomas Rustemeyer, Marie L. A. Schuttelaar, Suzanne G. M. A. Pasmans

**Affiliations:** 1 Department of Dermatology, Erasmus MC, University Medical Center, Rotterdam, The Netherlands; 2 Department of Dermatology, Center of Pediatric Dermatology, Sophia Children’s Hospital, Erasmus MC University Medical Center Rotterdam-Sophia Children’s Hospital, Rotterdam, The Netherlands; 3 Dutch Asthma Center Davos, Davos, Switzerland; 4 Swiss Institute of Allergy and Asthma Research (SIAF), University of Zürich, Davos, Switzerland; 5 Department of Dermatology, Sint Antonius Hospital, 3435 CM Nieuwegein, The Netherlands; 6 Microbiology and Systems Biology, TNO, Zeist, The Netherlands; 7 Department of Dermatology, University Medical Center Groningen, University of Groningen, Groningen, The Netherlands; 8 Dutch Patient Association for People with Atopic Dermatitis (VMCE: Vereniging voor Mensen met Constitutioneel Eczeem), Nijkerk, The Netherlands; 9 Department of Public Health, Erasmus MC, University Medical Center Rotterdam, Rotterdam, Netherlands; 10 Department of Pediatric Dermatology and Allergology, Wilhelmina Children’s Hospital, University Medical Center Utrecht, Utrecht, The Netherlands; 11 Department of Dermatology, Amsterdam University Medical Centres, Amsterdam, The Netherlands

**Keywords:** Atopic dermatitis, Therapeutic clothing, Antibacterial, Treatment, Topical corticosteroids, Randomized controlled trial

## Abstract

**Background:**

Atopic dermatitis (AD) is a chronic inflammatory skin disease that affects 10 to 20% of children and between 2 and 15% of the adults in Western Europe. Since 2000, therapeutic clothing or functional textiles based on silver or chitosan as antibacterial agents were introduced for AD. These agents aim to reduce skin colonization with *Staphylococcus* (*S*.) *aureus*. Increased colonization with *S. aureus* is correlated with increased AD severity. The antimicrobial effects of silver and chitosan have been demonstrated before. At this point, there is insufficient evidence for the effectiveness of antibacterial therapeutic clothing in patients with AD.

**Methods:**

This is a pragmatic randomized controlled double-blind multi-center trial comparing the effectiveness of antibacterial therapeutic clothing based on silver or chitosan as compared with non-antibacterial therapeutic clothing in patients with moderate to severe AD. A total of 165 participants, aged 0 to 80, diagnosed with moderate to severe AD are included. The study is performed in the Erasmus MC University Medical Center, University Medical Center Groningen, University Medical Center Utrecht, Amsterdam University Medical Centers, and St. Antonius Hospital Nieuwegein. Patients will be randomized 1:1:1 into one of the three intervention groups: group A will receive therapeutic clothing without antimicrobial agents, group B will receive microbial growth reducing therapeutic clothing based on chitosan, and group C will receive antimicrobial clothing based on silver. All therapeutic clothing is to be worn at night during the 12-month intervention period. Usual care is continued. The primary objective is to assess the effectiveness of antibacterial clothing (silver and chitosan group) as compared to non-antibacterial clothing assessed with the Eczema Area and Severity Index at 12 months compared to baseline. Secondary outcomes include between-group differences in physician- and patient-reported outcome measures, topical therapy use, *S. aureus* skin colonization, and safety. Data will be collected at baseline and after 1 month, 3 months, 6 months, and 12 months. A cost-effectiveness analysis will be performed.

**Discussion:**

This trial will provide data on the effectiveness, cost-effectiveness, and safety of antibacterial therapeutic clothing for patients with AD.

**Trial registration:**

ClinicalTrials.gov NCT04297215. Registered on 5 March 2020

**Supplementary Information:**

The online version contains supplementary material available at 10.1186/s13063-021-05836-y.

## Background

Atopic dermatitis (AD) is a chronic inflammatory skin disease that affects 10 to 20% of children in Western Europe [1]. About 70% of childhood AD will disappear spontaneously before adolescence and can be considered transient [2]. The prevalence among adults is estimated between 2 and 15%. Most patients are diagnosed with mild AD and are treated by a general practitioner. AD severity scores in Dutch general practices indicate that over 70% of pediatric patients have mild AD, while 28% have moderate and 2% severe disease [3].

AD treatment is aimed at control of inflammation, repair of the disrupted skin barrier with hydrating topical treatment, and avoidance of provoking factors. Anti-inflammatory drugs like corticosteroid creams and ointments are used to control inflammation. Emollients are used to improve hydration of the dry skin. Furthermore, therapeutic clothing has been used for decades as part of AD treatment. Historically, cotton bandages were used to cover the affected skin. This provides a fixation of creams and ointments, thereby possibly enhancing their action. It also protects the skin from further damage through scratching and irritating factors [4]. In the year 2000, therapeutic clothing or functional textiles based on silver or chitosan as antibacterial agents were introduced. The antibacterial properties of silver [5] and chitosan [6] have been demonstrated in healthcare before. In atopic dermatitis, antibacterial therapeutic clothing aims to reduce skin colonization with *Staphylococcus* (*S*.) *aureus*. Increased colonization with *S. aureus* is correlated with increased AD severity, and *S. aureus* induces further dysregulation of the inflammatory process [7, 8].

The effectiveness of (antibacterial) therapeutic clothing has been evaluated in several studies. A systematic review by the Dutch Society of Dermatologists (NVDV) concluded that there was insufficient evidence for the effectiveness from literature based on heterogeneity in treatments of small studies [9, 10]. However, both patients and clinicians had a positive experience with the use of antibacterial therapeutic clothing for the treatment of atopic dermatitis. Therefore, in the Dutch treatment guideline for AD, therapeutic clothing is advised in patients with moderate to severe dermatitis in whom a large surface area (>30%) is affected and those who suffer from severe itch, while treatment with topical corticosteroids cannot be tapered off [10]. More recently, the Dutch National Health Care Institute performed a systematic search on the same subject [11] and included two more studies [12, 13]. They concluded that, using the GRADE method for quality of evidence, there is low-quality evidence for the (cost-)effectiveness of silver-coated therapeutic clothing on the long term (90 days) and very low-quality evidence for the effectiveness on the short term (14 days to 4 weeks). Based on this report, the Dutch National Health Care Institute underlined the need for a trial to establish whether or not antibacterial therapeutic clothing is (cost-)effective for AD.

In this randomized controlled trial, we aim to investigate the (cost-)effectiveness of antibacterial therapeutic clothing (based on silver or chitosan) as compared to therapeutic clothing without these agents on reducing AD severity, thereby providing high-quality evidence to inform clinical practice.

## Methods

### Design and setting

The ABC study (AntiBacterial Clothing study) is a pragmatic, multi-center, double-blind, randomized controlled trial. The study aims to investigate the effectiveness of antibacterial therapeutic clothing based on silver or chitosan as compared with non-antibacterial therapeutic clothing in patients with moderate to severe AD. The study will be performed at the department of dermatology of the Erasmus MC in close collaboration with the departments of dermatology of the University Medical Center Groningen, University Medical Center Utrecht, Amsterdam University Medical Centers, and St. Antonius Hospital Nieuwegein. Both enrollment and the follow-up visits will take place at these five locations.

### Ethical considerations

The study follows the Dutch Medical Research Involving Human Subjects Act 1998 (WMO), the principles of the Helsinki Declaration 2008, and the European GDPR. All study procedures have been approved by the Institutional Review Board of the Erasmus MC University Medical Centre Rotterdam, The Netherlands (reference 2018-1609). Protocol amendments will be submitted for review at the Institutional Review Board.

### Participants

The study population consists of patients of all ages with moderate to severe AD according to the criteria of Williams [14]. Participants are eligible with an Eczema Area and Severity Index (EASI) score ≥ 6 at baseline [15]. Patients are not eligible if treated with therapeutic clothing, oral antibiotics, systemic immunosuppressive agents, or UV therapy until 1 month before inclusion or if treated with topical antibiotics within 1 week before inclusion. Other exclusion criteria are anamnestically assessed kidney function impairment, pregnancy or pregnancy wish, hypersensitivity to silver, or evidence of past non-compliance to treatments or appointments.

### Recruitment, inclusion, and consent

Patients are recruited from five hospitals across the country: Erasmus MC University Medical Center, University Medical Center Groningen, University Medical Center Utrecht, Amsterdam University Medical Centers, and St. Antonius Hospital. In addition, dermatologists in the Netherlands are informed about the study through the Dutch Society of Dermatology and Venereology, the Dutch Trial Network, and through scientific conferences. The patient support group (Vereniging voor Mensen met Constitutioneel Eczeem) will inform their members on this study, and online media will be used to inform potential participants. In addition, online media will be used to inform potential participants. After receiving information, potential participants can contact each site for additional information and assessment of eligibility. Eligibility is checked by a member of the research team and AD expert. These recruitment methods will enable us to recruit a representative sample of patients with moderate to severe AD across the Netherlands. Patients who are willing to participate and fulfill the inclusion criteria will be included in the study after providing written informed consent.

### Sample size

The null hypothesis of this study is that there is no difference between the antimicrobial therapeutic clothing (based on silver and chitosan) and therapeutic clothing without antimicrobial agents.

We used a standard deviation (*SD*) of 13 (based on the CLOTHES study [16]) and used the 6.6 minimal clinical important difference (MCID) for EASI as proposed by Schram et al. [17] resulting in an expected 0.51 Cohen’s *D* effect size. An alpha of 0.05 and a power of 0.80 were used.

Based on the inclusion of 3 groups, 5 measurements (baseline, 1 month, 3 months, 6 months, 12 months) and a 0.6 correlation between EASI scores (correlation is based on the CLOTHES study), while assuming a 20% loss to follow-up, we will include 55 patients per group and 165 patients in total.

### Randomization and blinding

**Fig. 1:**
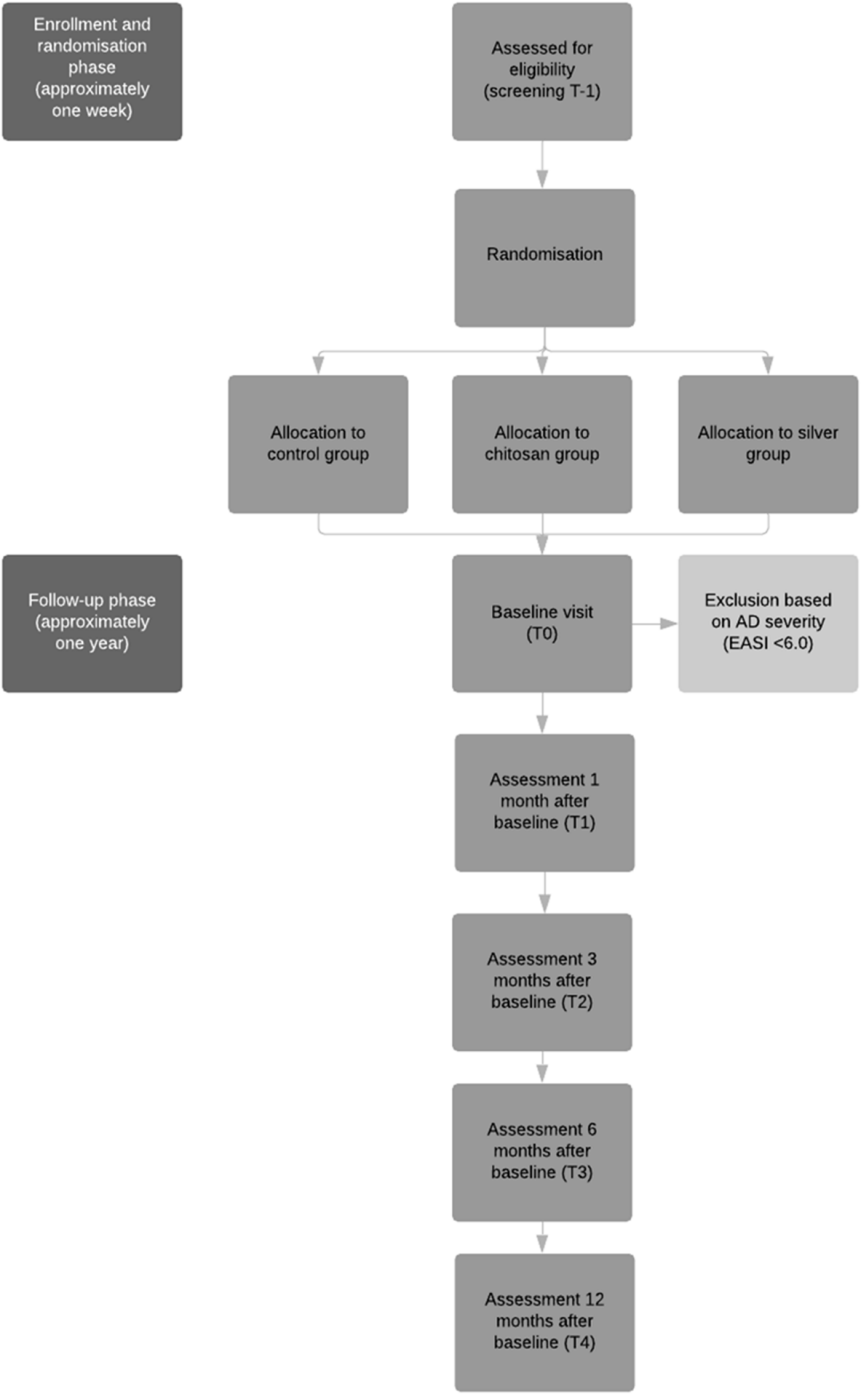
Flowchart of the study design

Randomization is carried out by the Erasmus MC University Medical Center. Included patients are stratified before randomization. Patients are stratified according to the severity in the strata “moderate dermatitis” (EASI 6.0–22.9) or “severe dermatitis” (EASI 23.0–72.0) [15] and according to age (0–5, 6–17, and >18 years). ALEA Clinical software will be used for randomization (random block size randomization, blocks of 3 and 6) [18]. An overview of the randomization procedure is provided in Fig. 1.

All labels and brand names are removed from the therapeutic clothing (“unlabeled”). In addition, the clothing will be provided by mail by a third party to ensure blinding.

Blinded efficacy assessors are unaware of treatment allocation. Furthermore, the type of therapeutic clothing is not described in the digital patient file and the treating clinician has no access to the CRFs until study completion.

### Intervention

Patients will be randomized into one of three intervention groups. Group A will receive therapeutic clothing without antimicrobial agents (control group), group B will receive microbial growth reducing therapeutic clothing based on chitosan, and group C will receive antimicrobial clothing based on silver.

The therapeutic clothing is to be worn at least at night during a 12-month intervention period. Participants will be encouraged to wear the clothing during each visit.

#### Therapeutic clothing

The control group will receive Binamed® therapeutic clothing without antimicrobial agents (BAP Medical). This is therapeutic clothing made of micro-modal and lycra. Micro-modal is a semi-synthetic wood cellulose fiber. This fiber has a high strength and elasticity and high moisture permeability.

The intervention groups receive either DermaCura® antimicrobial clothing (D&M) (group B) or Binamed antimicrobial therapeutic clothing (BAP Medical) (group C). DermaCura® antimicrobial clothing (D&M) is made from 98% TENCEL® C and 2% elastane. Chitosan (1%) is added to TENCEL ® C. Binamed antimicrobial therapeutic clothing (BAP Medical) consists of micro-modal lycra and woven silver filaments.

Patients will receive three sets of therapeutic clothing at the beginning of the study. During the study, three additional sets of therapeutic clothing can be requested by patients. Each set consists of a shirt with long sleeves and trousers. Socks and gloves can be prescribed if necessary. During the study, usual care (including the application of emollients, topical corticosteroid ointments, or creams only if needed and/or antihistamines) is continued according to the treatment guidelines of AD [10]. When AD worsens, the frequency and class of topical steroid treatment can be increased (as is usual care in the Netherlands). In case of a severe exacerbation requiring UV therapy or systemic treatment with immunosuppressive medication, participation in the study is stopped.

**Fig. 2:**
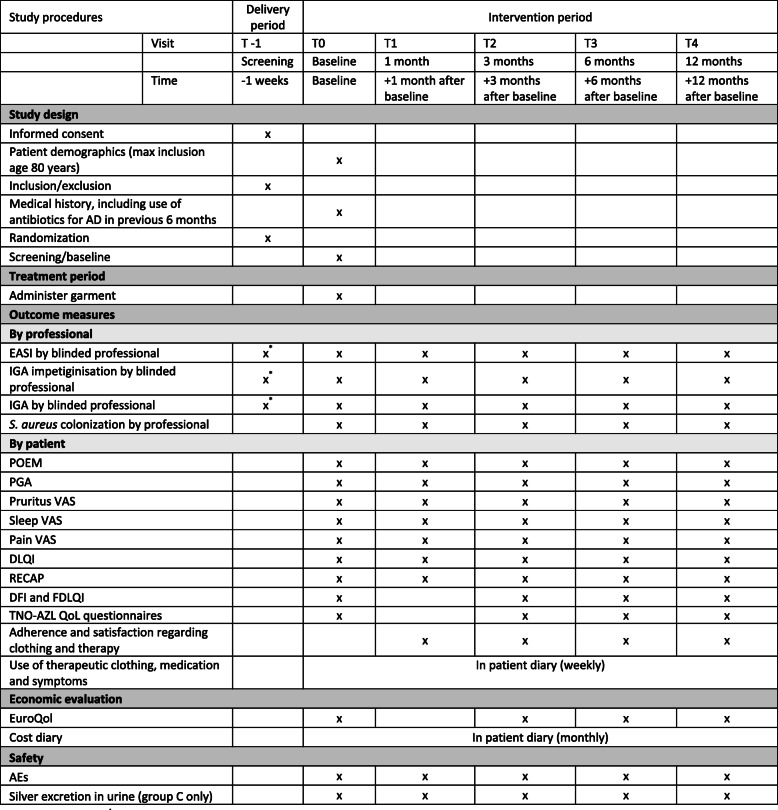
SPIRIT diagram of study procedures. *Optional

Measurements and assessments will be performed at baseline (start of intervention) and 1, 3, 6, and 12 months after baseline, as this reflects clinical practice. In addition, participants are requested to answer a weekly questionnaire on the use of therapy, AD symptoms, and costs. See Additional file 1 for an overview of the Standard Protocol Items: Recommendations for Interventional Trials (SPIRIT) 2013 checklist items. See Fig. 2 for the SPIRIT diagram of the trial procedures.

### Sample and laboratory procedures

In this study, bacterial swabs from the skin are obtained by a trained research assistant wearing non-sterile gloves. Sterile Copan MSwabs are used and samples will be collected for analysis of *S. aureus* colonization.

At the baseline assessment, swabs are taken from an affected part of the skin, preferably of the antecubital, popliteal, or neck fold. At all following visits, the swabs are taken from the same site. The skin surface is swabbed for 30 s.

Regarding the silver-coated textiles, there is a theoretical possibility for the adsorption of silver from the garment through the skin [19]. Therefore, silver excretion is assessed in urine in this group only. Morning urine of participants able to urinate in a urine cup will be collected and sent to a specialized laboratory. The urine of participants in the other groups will be collected as well in order to ensure that patients remain blinded.

### Primary outcome

The primary endpoint in this study is the difference in disease severity measured by the EASI [15] between the clothing without antimicrobial agents (control group) and microbial growth reducing/antimicrobial clothing based on chitosan or silver (intervention groups) at 12 months compared with baseline.

#### Secondary outcomes

Secondary outcomes include physician- and patient-reported effectiveness, degree of impetiginization, patient-reported quality of life, *S. aureus* colonization, use of topical corticosteroids and antibiotics, cost-effectiveness, and safety. Figure 2 provides an overview of the measurements per visit.

### Physician-reported outcomes

The effectiveness of the therapeutic clothing will be assessed by using the EASI and Investigator Global Assessment (IGA) [20] and IGA for impetiginization in atopic dermatitis. Eczema severity will be assessed by a blinded rater. EASI is a validated scale recommended by the Harmonising Outcome Measures for Eczema (HOME) initiative [21] as an outcome for eczema severity. The final EASI captures erythema, excoriation, edema, lichenification, and body surface area. The IGA impetiginization is an outcome measure for impetiginization in atopic dermatitis that still needs validation.

### Patient-reported outcomes

The Patient Orientated Eczema Measure (POEM) [22], pruritus visual analogue scale (VAS) [23], pain VAS, sleep disturbance VAS, and AD control measured by the recap of atopic eczema (RECAP) [24] questionnaire will be used as patient-reported outcome measures.

Health-related quality of life (HRQoL) for patients suffering from a skin disease will be measured by using the Dermatology Life Quality Index questionnaire (DLQI) [25] in adults, Children’s DLQI in children, and Infants’ DLQI in infants. The TNO-AZL quality of life questionnaires (Preschool Children’s Quality of Life, Children’s Quality of Life, and Adult Quality of Life) [26] and EuroQol questionnaires (EQ-5D-Y and EQ-5D-5L [27]) will be used to measure the overall quality of life.

In addition, the Dermatitis Family Impact (DFI) [28] and Family Dermatology Life Quality (FDLQI) [29] questionnaires will be used to assess the impact of AD on the patients’ family and/or partner and a specific questionnaire consisting of Likert and VAS scales will be used to assess patient satisfaction with the therapeutic clothing as treatment for AD.

### Adherence to therapeutic clothing

A weekly questionnaire will be used to evaluate the adherence to the therapeutic clothing.

### Topical corticosteroid use

The amount of topical corticosteroids (TCS) use will be derived from a weekly questionnaire. Participants are asked to fill out the number of days, frequency per day, and name of TCS that are used. The difference in the amount and TCS potency between groups will be compared as a marker for the effectiveness of the therapeutic clothing.

### *S. aureus* colonization

Skin colonization with *S. aureus* will be assessed as described earlier.

### Safety

Safety will be assessed by registration of adverse events and urinary silver excretion in the silver group as described in the laboratory procedures.

### Cost-effectiveness

The economic evaluation of the clothing without antimicrobial agents (control group) and microbial growth reducing/antimicrobial clothing based on chitosan or silver (intervention groups) will be calculated as the incremental cost-effectiveness ratio (ICER). The primary effect outcome measures will be the effectiveness of the therapeutic clothing assessed by using the EASI for the cost-effectiveness analysis (CEA) and quality-adjusted life years (QALY) for the cost-utility analysis (CUA). QALYs will be measured for a 1-year period, based on the Dutch tariff for the EQ-5D.

### Data collection, monitoring, and data analysis

ALEA will be used as a data management system [18]. Patient confidentiality will be ensured by using identification numbers. In a final stage, data from both management systems will be extracted to a SPSS dataset. Data management will be performed by the investigator and monitored by an independent monitor as predefined in the monitoring plan.

The data analysis will be conducted according to the intention-to-treat principle. The primary hypothesis is tested by means of linear mixed model analysis. The linear mixed model will use EASI as a dependent variable, and inclusion EASI, group, time, and time*group as independent variables; in addition, the EASI at baseline will be used as an additional covariate. As the time period between the visit varies (*t* = 1, 3, 6, and 12 months), time is defined as a continuous variable, in which we calculate the number of months from baseline. A two-sided alpha of 0.05 is used. Secondary outcome parameters will also be analyzed with linear mixed models. No interim analysis is planned, due to the low risks of participating in this study. Data will be available upon request.

## Discussion

The ABC study is a pragmatic randomized controlled multi-center trial that compares the effectiveness of antibacterial therapeutic clothing with non-antibacterial therapeutic clothing in patients with moderate to severe AD. This study will provide high-quality evidence to inform clinical practice whether or not antibacterial therapeutic clothing is effective for AD. In addition, the cost-effectiveness of antibacterial clothing will be evaluated.

This study will also provide useful insights in the treatment of AD during a longer period. Data concerning the use of topical corticosteroids, adherence to the clothing, and use of other therapy will be available for analysis. In addition, the fact that both children and adults will participate from five centers across the Netherlands will provide us with data that can be used to compare age differences and treatment differences across a semi-national level. In addition, this trial will be the first trial to monitor the safety of textiles with silver in patients with the abnormal skin barrier.

In conclusion, the ABC trial will assess the (cost-)effectiveness and safety of antibacterial therapeutic clothing with chitosan and silver over a treatment period of 12 months in patients with moderate to severe atopic dermatitis.

## Supplementary Information

**Additional file 1.** SPIRIT 2013 Checklist: Recommended items to address in a clinical trial protocol and related documents.

## Abbreviations

AD: Atopic dermatitis
EASI: Eczema Area and Severity Index
IGA: Investigator’s Global Assessment
POEM: Patient Orientated Eczema Measure
Pruritus VAS: Pruritus visual analogue scale
HRQOL: Health-related quality of life
*S*. *aureus*: *Staphylococcus aureus*
